# Error Analysis and Measurement Uncertainty for a Fiber Grating Strain-Temperature Sensor

**DOI:** 10.3390/s100706582

**Published:** 2010-07-09

**Authors:** Jaw-Luen Tang, Jian-Neng Wang

**Affiliations:** 1 Department of Physics, National Chung Cheng University, 168 University Road, Chia-Yi 62102, Taiwan; E-Mail: jawluen@phy.ccu.edu.tw; 2 Department of Construction Engineering, National Yunlin University of Science and Technology, 123 Section 3 University Road, Douliou 64002, Taiwan

**Keywords:** fiber Bragg grating, sensor, strain, temperature, error analysis, measurement uncertainty

## Abstract

A fiber grating sensor capable of distinguishing between temperature and strain, using a reference and a dual-wavelength fiber Bragg grating, is presented. Error analysis and measurement uncertainty for this sensor are studied theoretically and experimentally. The measured root mean squared errors for temperature T and strain ε were estimated to be 0.13 °C and 6 με, respectively. The maximum errors for temperature and strain were calculated as 0.00155 T + 2.90 × 10^−6^ ε and 3.59 × 10^−5^ ε + 0.01887 T, respectively. Using the estimation of expanded uncertainty at 95% confidence level with a coverage factor of *k* = 2.205, temperature and strain measurement uncertainties were evaluated as 2.60 °C and 32.05 με, respectively. For the first time, to our knowledge, we have demonstrated the feasibility of estimating the measurement uncertainty for simultaneous strain-temperature sensing with such a fiber grating sensor.

## 1. Introduction

The advantages of fiber optic sensors include light weight, small size, immunity to electromagnetic interference (EMI), large bandwidth, environmental ruggedness and electrical and optical multiplexing capability. Thus, fiber optic sensors are ideal for the applications in potential smart structures and materials. Fiber Bragg gratings (FBGs) have many wide applications, ranging from tele-communications to optical fiber sensors. Though Bragg grating sensors (BGSs) have offered a variety of potential advantages over their conventional counterparts, their widespread practical use has been plagued by their inability to effectively discriminate between temperature and strain fields. A number of attempts to overcome this limitation have been demonstrated [1–7]. Among them, one popular approach is to use a separate reference grating as a temperature sensor. Another popular approach is the dual-wavelength technique which involves writing two superimposed Bragg gratings, resulting in different responses to temperature and strain at the same location. Therefore, the combination of the respective merits of both reference grating and dual-wavelength grating techniques is expected to offer excellent strain and temperature performance. Chehura *et al.* have used a technique which exploits the core-cladding mode coupling of a tilted fibre Bragg grating (TFBG). The strength of this method lays in the use of only a single TFBG, and the wavelength and matrix induced errors for temperature and strain measurements are 1 °C and 11 με, respectively [8]. Ma *et al*. have presented an error analysis of temperature-compensated white-light interferometric fiber-optic strain sensor. The theoretical and experimental analysis demonstrates its potential for practical applications. For example, 4 m sensing and 6 m compensating fibers using Fibercore HB800 fiber can enhance the strain resolution to 2.5 με and reduce the temperature compensating error to ±7 με [9]. However, the principle of this sensor is not based on the concept of fiber grating. Xie *et al.* have analyzed the relationship between temperature sensitivity and plating thickness of nickel-clad FBG in theory and verified by experiment. The rectangular distribution is used to calculate the standard uncertainty, *u_s_*, and the expanded uncertainty of temperature is obtained: *U* = *ku_s_* = 0.28 pm °C^−1^, where *k* = 2 at a level of confidence of 95% [10]. In this paper, we present an evaluation of error analysis and measurement uncertainty for a reference dual-wavelength grating sensor system. The theoretical strain and temperature dependent errors, both wavelength and matrix induced errors, for the grating pair were examined. The measurement uncertainty for simultaneous temperature and strain measurements using a reference dual wavelength grating method was studied by an estimation of standard uncertainty, combined standard uncertainty, and expanded uncertainty. To our knowledge, this is the first time that the measurement uncertainty for simultaneous strain-temperature sensing was demonstrated for fiber grating sensors.

## 2. Sensor Description and Operation Principle

### 2.1. Simultaneous Temperature and Strain Measurement

Though BGSs have offered potentially numerous advantages over their conventional electrical and mechanical counterparts, their widespread use has been limited by their inability to discriminate effectively between temperature and strain fields, and this poses serious problems for sensors system designed to monitor quasi-strain signals, as temperature variations along the fiber link will induce indistinguishable thermal-apparent strain signals. It is apparent that measurement of one wavelength shift from a single grating will not determine those two variables simultaneously. A number of methods that separate temperature- and strain-induced wavelength shift and overcome this limitation have been proposed and demonstrated, including the use of reference grating [1], the use of dual wavelength gratings [2], and the use of two sensors associated with different strain and temperature responses [3–7,11]. In brief, the determination of those two variables or the elimination of cross-sensitivity may be achieved by operating at two wavelengths or two different perturbation-induced optical modes, which have different responses to temperature and strain. The simplest scheme is to facilitate two sensors in which one is isolated from either of the unwanted external perturbations. One approach called reference grating is to use separate reference gratings as temperature sensors along the fiber path, *i.e.,* gratings that are in thermal contact with the local structure but shield from strain changes. Another approach is to use the dual wavelength technique involving writing two superimposed Bragg gratings [2], in which the responses to temperature (κ_1T_,κ_2T_) and strain (κ_1ε_,κ_2ε_) at the same location on the structure are different. Dual-wavelength technique requires two broadband sources to address each sensor and suitable wavelength demodulation system (WDS) at the output. The change in the Bragg center wavelengths Δλ_i_ of the two gratings from the changes in temperature (ΔT_i_) and strain (Δε_i_) is given by the following matrix expression

(1) $$\Delta \lambda_{i}=\kappa_{i\varepsilon }\Delta \varepsilon_{i}+\kappa_{\mathrm{iT}}\Delta T_{i}\;i=1,2$$

where κ*_i_*_ε_ = *∂*λ / *∂*ε*_i_* is the strain coefficient of material related to the Poisson ratio, photoelastic constant and effective refractive index, and κ*_iT_* = *∂*λ / *∂T_i_* is the temperature coefficient related to the thermal expansion and thermo-optic coefficients. The above matrix can be inverted to give temperature and strain provided that the ratio of temperature responses of the two gratings is different from that of their strain responses. Xu *et al.* [2] have used this method to measure the responses of two BGS’s written at 848 and 1,298 nm, and reported that the responses are 6.5% higher for strain and 9.8% less for temperature for wavelength at 1300 nm compared with 850 nm. This approach has shown the capability to measure strain and temperature simultaneously with errors of ±10 με and ±5 °C, respectively. Kannellopoulos *et al.* [3] have demonstrated simultaneous temperature and strain measurement using a FBG and a long-period rocking filter operating at the 800 nm wavelength band and reported errors of ±165 με and ±1.5 °C. Other similar types of Bragg gratings, such as a long-period grating and FBG’s in the 1,300 nm band, demonstrated by Patrick *et al.* [4], can be used to determine strain and temperature of ±9 με and ±1.5 °C, respectively. However, several potential problems such as relative large bandwidth and long physical length of long-period grating, have limited the accuracy and number of sensors that could be used in a wavelength division multiplexing (WDM) system. If spatial resolutions can be improved to a certain degree, this technique could provide a practical means for measuring strain/temperature in arrays of distributed sensor systems.

### 2.2. Sensor Configuration

**Figure 1.**
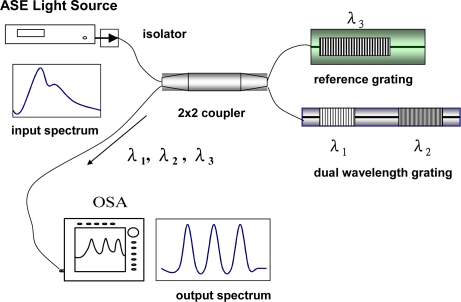
A reference dual wavelength grating system.

We have developed a simple and low-cost optical fiber sensor for this purpose [12]. Table 1 summarizes the experimental and theoretical errors of individual strain and temperature measurement. Figure 1 shows the configuration of the sensor and the detection system, in which the proposed sensor was connected to the output port of a fiber coupler. The fiber sensor was consisted of a bare grating pair (λ_1_ and λ_2_,) and a packaged reference grating (λ_3_). The bare grating pair was constructed by fusion splicing two fiber Bragg gratings in cascade with different Bragg wavelengths. The spliced portion of the grating pair was glued into a quartz tube in order to prevent the relatively brittle spliced or fused portion from being damaged or broken. To protect the reference grating from mechanical deformation and damage, a method of packaging the bare fiber Bragg grating with a stainless steel tube was applied. In the packaging process, the reference grating was first bonded to a quartz substrate with an adhesive and the substrate with the reference grating was inserted into a stainless steel tube, and the both ends of the stainless tube was then glued and sealed with elastic epoxy glue. In this sensor structure, the free end of the fiber was secured with adhesive tape to avoid any unwanted movement or twisting. The three fiber Bragg gratings at wavelengths of λ_1_, λ_2_, λ_3_ were interrogated using a broadband ASE source and an optical spectrum analyzer (OSA). A fiber coupler was used for coupling the reflected light signals of the sensor to the OSA. The reference grating was used to measure only the temperature effect. The shift in Bragg wavelength λ_3_ from temperature changes is given by

(2) $$\Delta \lambda_{3}=\kappa_{3T}\Delta T$$

Precise measurement of wavelength shift Δλ_3_ can be used to determine uniquely the local temperature provided that the temperature coefficient κ_3T_ is well known. The grating pair was fabricated by splicing two fiber Bragg gratings with wavelengths, λ_1_ and λ_2_, respectively. The wavelength shifts Δλ_i_ from temperature (ΔT_i_) and strain (Δε_i_) changes were calculated using Equation (1). This equation may be inverted and temperature and strain from measurements of the two wavelength shifts can be solved as:

(3) $$\left(\begin{array}{c} \Delta T\\ \Delta \varepsilon \end{array}\right)=\frac{1}{\left(\kappa_{1T}\kappa_{2\varepsilon }-\kappa_{2T}\kappa_{1\varepsilon }\right)}\left(\begin{array}{cc} \kappa_{2\varepsilon } & -\kappa_{1\varepsilon }\\ -\kappa_{2T} & \kappa_{1T} \end{array}\right)\left(\begin{array}{c} \Delta \lambda_{1}\\ \Delta \lambda_{2} \end{array}\right)$$

**Figure 2.**
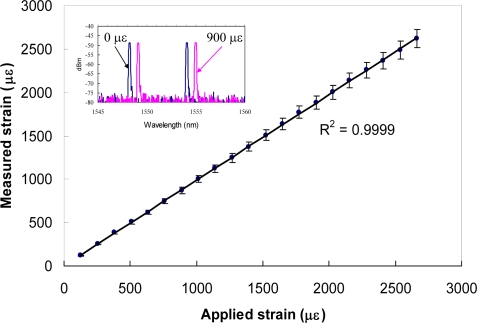
Strain performance of grating sensor. Inset: output spectrum for the fiber Bragg grating sensors with an applied strain at 0 με (black color) and 900 με (magenta color), respectively.

**Figure 3.**
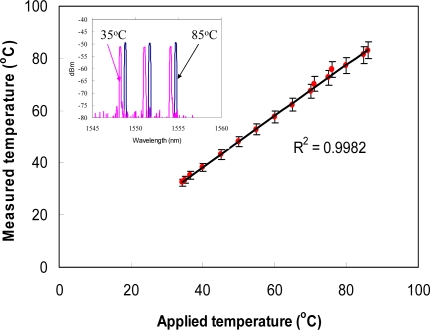
Temperature performance of grating sensor. Inset: output spectrum for the fiber Bragg grating sensors with an applied temperature at 35 °C (black color) and 85 °C (magenta color), respectively.

**Figure 4.**
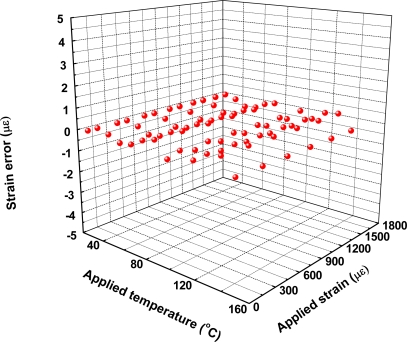
3D Scatter plot of strain errors with applied temperature and applied strain.

**Figure 5.**
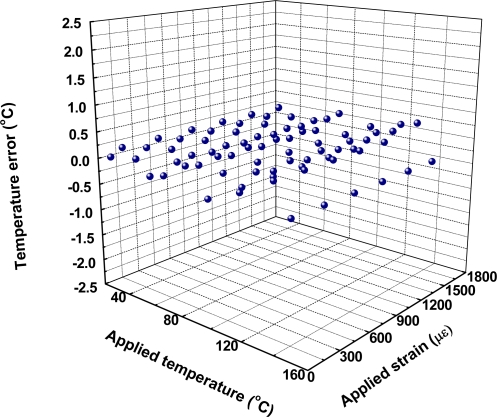
3D scatter plot of temperature errors with applied temperature and applied strain.

It can be seen that the errors measured in temperature and strain are determined primarily by the resolution effect of optical spectrum analyzer and the errors in estimation of temperature and strain coefficients (see Table 1, κ_1ε_ = 0.914 pm/με; κ_2ε_ = 0.918 pm/με; κ_1T_ = 10.4 pm/°C; κ_2T_ = 12.1 pm/°C). Although the use of a fast and high resolution grating interrogation system is feasible, to build such a sensor system is costly. A simple and cost-effective method for improving the performance is to use the reference grating as an independent temperature sensor. The reference grating can be used to reduce unnecessary errors induced from the grating pair and to improve the accuracy of the temperature measurement. Figures 2 and 3 show the strain and temperature performance of an individual fiber Bragg grating sensor. Figures 4 and 5 show the results of simultaneous strain and temperature measurements for this reference dual-wavelength sensor. The measured root mean square (RMS) errors for temperature and strain were estimated to be 0.13 °C and 6 με, respectively.

## 3. Error Analysis of Fiber Grating Sensor

According to the error analysis technique presented by Jin *et al.* [13], the theoretical strain and temperature dependent errors for the grating pair were examined. In the first case, we neglected the errors of the measured coefficients and attributed all errors to measurement errors of λ_1_ and λ_2_. The maximum errors in temperature T and strain ε is formulated as:

(4) $$\left|\delta T\right|\leq \frac{\left|\kappa_{2\varepsilon }\right|\left|\delta \lambda_{1}\right|+\left|\kappa_{1\varepsilon }\right|\left|\delta \lambda_{2}\right|}{\left|\Delta \right|}$$

(5) $$\left|\delta \varepsilon \right|\leq \frac{\left|\kappa_{2T}\right|\left|\delta \lambda_{1}\right|+\left|\kappa_{1T}\right|\left|\delta \lambda_{2}\right|}{\left|\Delta \right|}$$

where Δ = κ_1T_κ_2ε_ − κ_2T_κ_1ε_. The maximum measurement errors of δT and δε were calculated as 0.13 °C and 1.6 με, respectively. For the second case, assuming that the measurement errors in λ_1_ and λ_2_ may be neglected (δλ_1_ = δλ_2_ = 0) and the maximum errors are in all the coefficients, the maximum relative errors for δT/T and δε/ε is expressed as:

(6) $$\left|\frac{\delta T}{T}\right|\text{max}\approx \frac{\left|\kappa_{1T}\kappa_{2ɛ}\delta_{1T}\right|+\left|\kappa_{1ɛ}\kappa_{2T}\delta_{2T}\right|+\left|\kappa_{1ɛ}\kappa_{2ɛ}\right|\left(\left|\delta_{1ɛ}\right|+\left|\delta_{2ɛ}\right|\right)\left|ɛ/T\right|}{\left|\Delta \right|}$$

(7) $$\left|\frac{\delta ɛ}{ɛ}\right|\text{max}\approx \frac{\left|\kappa_{1ɛ}\kappa_{2T}\delta_{1ɛ}\right|+\left|\kappa_{1T}\kappa_{2ɛ}\delta_{2ɛ}\right|+\left|\kappa_{1T}\kappa_{2T}\right|\left(\left|\delta_{1T}\right|+\left|\delta_{2T}\right|\right)\left|T/ɛ\right|}{\left|\Delta \right|}$$

**Figure 6.**
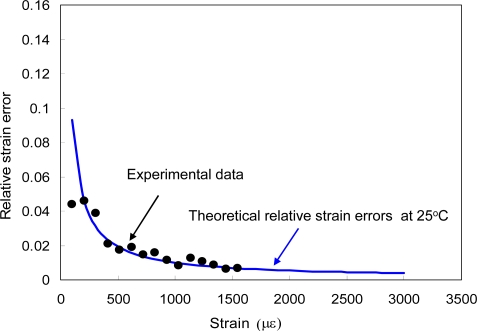
Measured and calculated relative strain error as a function of strain. In error analysis, the measurement errors in wavelength were neglected.

Thus, the maximum relative errors for δT/T and δε/ε were calculated as 0.0016 + 2.90 × 10^−6^ ε/T, and 3.59 × 10^−5^ + 0.0188 T/ε, respectively. In Figure 6, we plotted the experimental relative error δε/ε as a function of strain at room temperature (25 °C) along with a theoretical curve at 25 °C. It can be seen that the theoretical curve and the experimental data of the relative strain errors agreed well except for some measured data below 200 με, indicating that the error in wavelength measurement is not negligible.

**Figure 7.**
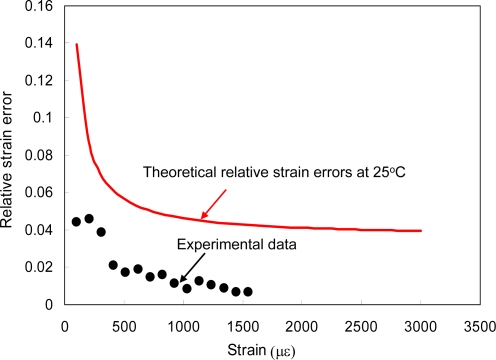
Measured and calculated relative strain error as a function of strain. In error analysis, all measurement errors are taken into account.

Secondly, considering all the measurement errors involved in determining the coefficients and the precision of wavelength measurement, the maximum relative errors for δT/T and δε/ε is given by

(8) $${\left|\frac{\delta T}{T}\right|}_{\text{max}}\approx \frac{\left|\kappa_{1T}\kappa_{2\varepsilon }\right|+\left|\kappa_{1\varepsilon }\kappa_{2T}\right|+2\left|\varepsilon /T\right|\left|\kappa_{1\varepsilon }\kappa_{2\varepsilon }\right|}{\left|\kappa_{1T}\kappa_{2\varepsilon }-\kappa_{1\varepsilon }\kappa_{2T}\right|}\left|\gamma \right|$$

(9) $${\left|\frac{\delta \varepsilon }{\varepsilon }\right|}_{\text{max}}\approx \frac{\left|\kappa_{1\varepsilon }\kappa_{2T}\right|+\left|\kappa_{1T}\kappa_{2\varepsilon }\right|+2\left|T/\varepsilon \right|\left|\kappa_{1T}\kappa_{2T}\right|}{\left|\kappa_{1T}\kappa_{2\varepsilon }-\kappa_{1\varepsilon }\kappa_{2T}\right|}\left|\gamma \right|$$

where γ is the maximum error in the entire matrix. With Equations (8) and (9), the maximum relative error or δT/T and δε/ε are estimated as 0.00175 + 1.42 × 10^−4^ ε/T, and 1.75 × 10^−3^ + 0.021 T/ε, respectively. As shown in Figure 7, the experimental error of δε / ε as a function of strain was plotted together with the different maximum error predictions at 25 °C. It is shown that all the measured data in Figure 7 departed away from the theoretical curve by a factor of 1.8∼6.2. Since the theoretical curve represents the maximum relative errors, the results show that all of the measured data of the relative strain errors were well controlled within the theoretical calculations at a reasonable range, in which the differences of relative strain errors between experiment and theory were around 0.03∼0.09.

However, in Reference [13], equations (6) to (9) were used only for normalizing maximum error, δT_max_ or δε_max_, to be relative maximum error (δT/T or δε/ε, unit less). The value of relative maximum error (percentage) is not always between 0 and 100. It is not recommended to use the relative maximum error when the temperature or strain is zero since relative maximum error becomes singular in this situation. Actually, for our laboratory testing data, the controlled temperature and strain ranges were 25∼113 °C and 100∼1,600 με, respectively. There was no singular problem for our testing results. Therefore, using maximum errors instead of relative maximum errors could be a better way to characterize measured quantity. The maximum errors for temperature and strain are expressed by

(10) $${\left|\delta T\right|}_{\text{max}}\approx \frac{(\left|\kappa_{1T}\kappa_{2ɛ}\delta_{1T}\right|+\left|\kappa_{1ɛ}\kappa_{2T}\delta_{2T}\right|)\left|T\right|+\left|\kappa_{1ɛ}\kappa_{2ɛ}\right|\left(\left|\delta_{1ɛ}\right|+\left|\delta_{2ɛ}\right|\right)\left|ɛ\right|}{\left|\Delta \right|}$$

(11) $${\left|\delta ɛ\right|}_{\text{max}}\approx \frac{(\left|\kappa_{1ɛ}\kappa_{2T}\delta_{1ɛ}\right|+\left|\kappa_{1T}\kappa_{2ɛ}\delta_{2ɛ}\right|)\left|ɛ\right|+\left|\kappa_{1T}\kappa_{2T}\right|\left(\left|\delta_{1T}\right|+\left|\delta_{2T}\right|\right)\left|T\right|}{\left|\Delta \right|}$$

With equations (8) and (9), the maximum errors, |δT|_max_ and |δε|_max_, were estimated as 0.00155 T + 2.90 × 10^−6^ ε and 3.59 × 10^−5^ ε+ 0.01887 T, respectively.

## 4. Measurement Uncertainty of Fiber Grating Sensor

The measurement uncertainty for temperature and strain simultaneous measurements using dual wavelength grating method was studied as estimation of standard uncertainty, combined standard uncertainty, and expanded uncertainty [14,15]. The source of uncertainty includes the skills of operators, effects of broadband ASE light source stability, fabrication and preparation of fiber grating samples and the resolution of optical spectrum analyzer. Assuming the operators are well-trained, broadband light source has been calibrated and is in stable condition, and the fabrication of fiber grating sensor meets the allowable tolerances as specified in standards or methods.

### 4.1. Estimation of Standard Uncertainty

The models for temperature and strain difference were shown in Equation (3) and let functions *f* and *g* represent the temperature and strain differences:

(12) $$f\underset{˙}{=}\Delta T=[\kappa_{2\varepsilon }\left(\Delta \lambda_{1}\right)-\kappa_{1\varepsilon }\left(\Delta \lambda_{2}\right)]/\left[\kappa_{1T}\kappa_{2\varepsilon }-\kappa_{2T}\kappa_{1\varepsilon }\right]$$

(13) $$g=\Delta \varepsilon =\left[-\kappa_{2T}\left(\Delta \lambda_{1}\right)+\kappa_{1T}\left(\Delta \lambda_{2}\right)\right]/\left[\kappa_{1T}\kappa_{2\varepsilon }-\kappa_{2T}\kappa_{1\varepsilon }\right]$$

Since the measurement resolution of wavelength shift using the ANDO AQ6331 OSA was ±0.05 nm, the uncertainty on the FBGs measurement was as large as 3 pm [16]. For the standard uncertainty of wavelength shift at a confidence level of not less than 95%; assuming normal distribution with coverage factor *k* = 2 and degree of freedom, ν_Δλ1_ = infinity, and ν_Δλ2_ = infinity, therefore;

Standard uncertainty, u_Δλ1_ *=* 3 pm/2 = 1.5 pm;
Standard uncertainty, u_Δλ2_ = 3 pm/2 = 1.5 pm;

### 4.2. Estimation of Combined Standard Uncertainty

Based on Table 1, the strain and temperature coefficients were used to evaluate the uncertainty values for both fiber gratings, λ_1_ and λ_2_, respectively.

Sensitivity coefficient for Δ T due to λ_1_:
  $$C_{f,\Delta \lambda 1}=\left[\partial f/\partial_{\Delta \lambda 1}\right]=\kappa_{2\varepsilon }/\left[\kappa_{1T}\kappa_{2\varepsilon }-\kappa_{2T}\kappa_{1\varepsilon }\right]=0.6070626\;{}^{\circ}C/\text{pm};$$
Sensitivity coefficient for Δ T due to λ_2_:
  $$C_{f,\Delta \lambda 2}=\left[\partial f/\partial_{\Delta \lambda 1}\right]\tilde{=}\;\kappa_{1\varepsilon }/\left[\kappa_{1T}\kappa_{2\varepsilon }-\kappa_{2T}\kappa_{1\varepsilon }\right]=-0.6044174\;{}^{\circ}C/\text{pm};$$
Sensitivity coefficient for Δε due to λ_1_:
  $$C_{g,\Delta \lambda 1}=\left[\partial g/\partial_{\Delta \lambda 1}\right]\tilde{=}\kappa_{2T}/\left[\kappa_{1T}\kappa_{2\varepsilon }-\kappa_{2T}\kappa_{1\varepsilon }\right]=-8.001587\;\mu \varepsilon /\text{pm};$$
Sensitivity coefficient for Δε due to λ_2_:
  $$C_{g,\Delta \lambda 2}=\left[\partial g/\partial_{\Delta \lambda 1}\right]=\;\kappa_{1T}/\left[\kappa_{1T}\kappa_{2\varepsilon }-\kappa_{2T}\kappa_{1\varepsilon }\right]=-6.8773972\;\mu \varepsilon /\text{pm};$$

Since the temperature is compensated, it is reasonable to assume there are non-correlated uncertainty components. The combined uncertainty is obtained from the uncertainties of the single components without taking into account possible covariances. The combined uncertainties for temperature and strain are the square root of equations (14) and (15), respectively:

(14) $$U_{\mathrm{combined}}^{2}\left(\Delta T\right)=u_{f}=\sum_{i=1}^{n}{\left(\frac{\partial f}{\partial x_{i}}\right)}^{2}u^{2}\left(x_{i}\right)$$

(15) $$U_{\mathrm{combined}}^{2}\left(\Delta \varepsilon \right)=u_{g}=\sum_{i=1}^{n}{\left(\frac{\partial g}{\partial x_{i}}\right)}^{2}u^{2}\left(x_{i}\right)$$

where the combined uncertainty is calculated for non-correlated uncertainty components based on the first order Taylor approximation.

Thus the combined standard uncertainty for temperature difference as:

$$\begin{array}{lll} {\left[u_{f}\right]}^{2} & = & {\left(C_{f,\Delta \lambda 1}\right)}^{2}{\left(u_{\Delta \lambda 1}\right)}^{2}+{\left(C_{f,\Delta \lambda 2}\right)}^{2}{\left(u_{\Delta \lambda 2}\right)}^{2}=1.651\;{}^{\circ}C^{2};\\ & & u_{f}=1.285{}^{\circ}C \end{array}$$

The combined standard uncertainty for strain difference as:

$$\begin{array}{lll} {\left[u_{g}\right]}^{2} & = & {C_{g,\Delta \lambda 1})}^{2}{\left(u_{\Delta \lambda 1}\right)}^{2}+{\left(C_{g,\Delta \lambda 2}\right)}^{2}{\left(u_{\Delta \lambda 2}\right)}^{2}=250.479\;{\mu \varepsilon }^{2};\\ & & u_{g}=15.827\;\mu \varepsilon \end{array}$$

### 4.3. Estimation of Expanded Uncertainty

Effective degree of freedom for Δ T,

$$\nu_{eff,f}={\left[u_{f}\right]}^{4}/\Sigma \{{\left[(C_{f,\Delta \lambda t})(u_{\Delta \lambda t})\right]}^{4}/\nu_{i}\}=\text{infinity};$$

Effective degree of freedom for Δε,

$$\nu_{\mathrm{eff},g}={\left[u_{g}\right]}^{4}/\sum \left\{{\left[(C_{g,\Delta \lambda \iota })(u_{\Delta \lambda \iota })\right]}^{4}/\nu_{i}\right\}=\text{infinity};$$

Thus for coverage factor *k* = 2.025 at 95% level confidence (from *Student-t* distribution);

$$U_{f}=U_{\Delta T}=k\;\;u_{f}=2.205*(1.285)≅2.602{}^{\circ}C$$

$$U_{g}=U_{\Delta \varepsilon }=k\;\;u_{g}=2.205*(15.827)≅32.049\;\mu \varepsilon$$

Therefore, values of temperature and strain measurement uncertainty were determined to be 2.602 °C and 32.049 με, respectively. The estimation of expanded uncertainty provides at 95% confidence level with a coverage factor of *k* = 2.205, but excluding the effects of light source stability and fabrication and preparation of fiber grating samples.

## 5. Conclusions

We present a simple and low-cost reference dual-wavelength grating sensor system that could offer the potential of simultaneous measurement of strain and temperature for infrastructures. Experimental results show that measurement errors of 6 με and 0.13 °C for strain and temperature could be achieved, respectively. We have performed and characterized the error analysis and measurement uncertainty for this strain-temperature sensing system. The maximum errors for temperature T and strain ε were calculated as 0.00155 T + 2.90 × 10^−6^ ε and 3.59 × 10^−5^ ε+ 0.01887 T, respectively. Based on the analysis of estimation of expanded uncertainty at 95% confidence level with a coverage factor of *k* = 2.205, values of temperature and strain measurement uncertainty were evaluated as 2.60 °C and 32.05 με, respectively. Using fiber grating sensors, for the first time the measurement uncertainty for simultaneous strain-temperature sensing could successfully be analyzed.

## Figures and Tables

**Table 1.** Experimental and theoretical errors of individual strain and temperature measurement.

**FBG sensor property**	**Dual grating**		**Reference grating**
	***λ*_1_^1^ (1,548 nm)**	***λ*_2_^1^ (1,554 nm)**	***λ*_3_ (1,551 nm)**
Strain coefficient (pm/με)	0.914 ± 0.003	0.918 ± 0.003	N/A^2^
Temperature coefficient (pm/°C)	10.4 ± 0.10	12.1 ± 0.10	12.1 ± 0.08
Theoretical strain error (με)	5.36	4.93	N/A
Experimental strain error (με)	7.86	12.35	N/A
Theoretical temperature error (°C)	0.17	0.17	0.17
Experimental temperature error (°C)	0.65	0.44	0.48

Note:

1 Strain and temperature coefficients for calculation of measurement uncertainty were as follows: κ_1ε_ = 0.914 pm/με; κ_1ε_ = 0.918 pm/με; κ_1T_ = 10.4 pm/°C; κ_2T_ = 12.1 pm/°C

2 Not applicable
